# Apico-basal Polarity Determinants Encoded by *crumbs* Genes Affect Ciliary Shaft Protein Composition, IFT Movement Dynamics, and Cilia Length

**DOI:** 10.1534/genetics.117.300260

**Published:** 2017-09-07

**Authors:** Khodor Hazime, Jarema J. Malicki

**Affiliations:** Bateson Centre, Department of Biomedical Science, University of Sheffield, S10 2TN, United Kingdom

**Keywords:** cilia, Crumbs, cristae, IFT, apico-basal polarity

## Abstract

One of the most obvious manifestations of polarity in epithelia is the subdivision of the cell surface by cell junctions into apical and basolateral domains. *crumbs* genes are among key regulators of this form of polarity. Loss of *crumbs* function disrupts the apical cell junction belt and *crumbs* overexpression expands the apical membrane size. Crumbs proteins contain a single transmembrane domain and localize to cell junction area at the apical surface of epithelia. In some tissues, they are also found in cilia. To test their role in ciliogenesis, we investigated mutant phenotypes of zebrafish *crumbs* genes. In zebrafish, mutations of three *crumbs* genes, *oko meduzy*/*crb2a*, *crb3a*, and *crb2b*, affect cilia length in a subset of tissues. In *oko meduzy* (*ome*), this is accompanied by accumulation of other Crumbs proteins in the ciliary compartment. Moreover, intraflagellar transport (IFT) particle components accumulate in the ciliary shaft of *ome*;*crb3a* double mutants. Consistent with the above, *Crb3* knockdown in mammalian cells affects the dynamics of IFT particle movement. These findings reveal *crumbs*-dependent mechanisms that regulate the localization of ciliary proteins, including Crumbs proteins themselves, and show that *crumbs* genes modulate intraflagellar transport and cilia elongation.

Cilia are finger-like cell surface protrusions that house components of many signal transduction cascades (Schou *et al.* 2015; Mourão *et al.* 2016; Malicki and Johnson 2017). The detection of photons by photoreceptors and chemicals by olfactory sensory neurons is mediated by signal transduction mechanisms inside the ciliary shaft (Jenkins *et al.* 2009; Kennedy and Malicki 2009). Vertebrate hedgehog signaling requires cilia and wnt, the platelet-derived growth factor and mTOR pathways are modulated by them [reviewed in Schou *et al.* (2015), Mourão *et al.* (2016), Malicki and Johnson (2017)]. In addition to signaling functions, cilia have a hydrodynamic role: their movement drives the flow of fluid in ducts and vesicles, such as the pronephric duct in zebrafish or the embryonic node in the mouse (Kramer-Zucker *et al.* 2005; Hirokawa *et al.* 2012). They also propel cells, such as sperm cells.

In cells that display apico-basal polarity, almost without exception, cilia form at the apical surface. Consequently, the ciliary membrane is an apical surface subcompartment, characterized by a unique protein and lipid content (Craige *et al.* 2010; Hu *et al.* 2010; Mukhopadhyay *et al.* 2010; Chih *et al.* 2011). Ciliated cells of epithelial sheets thus feature two cell membrane subdivisions: the one that separates the apical and basolateral domains and another one that separates the ciliary membrane from the rest of the apical surface. *crumbs* genes were initially discovered as essential regulators of the apico-basal cell membrane subdivision in fly embryonic epithelia (Jurgens *et al.* 1984; Tepass *et al.* 1990; Wodarz *et al.* 1995). They encode transmembrane (TM) proteins that localize to the vicinity of epithelial cell junctions, feature a short cytoplasmic tail and an extracellular domain of varying size (Tepass *et al.* 1990; van den Hurk *et al.* 2005; Omori and Malicki 2006). Loss of *crumbs* function in the fly disrupts the cell junction belt at the boundary of the apical and the basolateral surface, and *crumbs* overexpression expands apical membrane size (Wodarz *et al.* 1995; Grawe *et al.* 1996). A similar function of *crumbs* genes has been observed in vertebrates; mutations in one of the zebrafish *crumbs* loci, *oko meduzy*, and the locus encoding a related apico-basal polarity determinant, *nagie oko*, a fly *stardust* homolog, cause loss of apical–basal polarity in the eye neuroepithelium and a severe neuronal patterning defect in the retina (Malicki *et al.* 1996; Malicki and Driever 1999; Wei and Malicki 2002; Omori and Malicki 2006). A related *crumbs* function in apico-basal polarity is also evident in fly and zebrafish photoreceptor cells (Pellikka *et al.* 2002; Hsu *et al.* 2006; Omori and Malicki 2006). Finally, while the apico-basal polarity function is mostly mediated by its intracellular tail (Wodarz *et al.* 1995), Crumbs extracellular domains mediate cell adhesion in the zebrafish photoreceptor cell layer (Zou *et al.* 2012) and human CRB1 mutations cause severe, early-onset retinal degeneration (den Hollander *et al.* 1999).

Vertebrate Crumbs and related apico-basal polarity determinants also affect cilia formation. While one *crumbs* gene exists in the fly, the human and zebrafish genomes contains three and five *crumbs* genes, respectively (van den Hurk *et al.* 2005; Omori and Malicki 2006; Gosens *et al.* 2008). Zebrafish *crumbs* genes display distinct expression patterns. *crb2b*, for example, is highly enriched in the pronephros and in photoreceptor cells (Hsu *et al.* 2006; Omori and Malicki 2006; Zou *et al.* 2012). *crb3a*, on the other hand, is expressed predominantly in the otic vesicle at stages that were investigated thus far (Omori and Malicki 2006). Consistent with these expression patterns, antisense morpholino knockdown of zebrafish *crb2b* and *crb3a* reduces cilia size in the pronephros and the ear, respectively (Omori and Malicki 2006). An even stronger *crumbs* phenotype has been reported in tissue culture; small interfering RNA (siRNA) knockdown of the *crumbs 3* gene in Madin-Darby canine kidney (MDCK) cells eliminates cilia entirely (Fan *et al.* 2004). In agreement with *crumbs* cilia phenotype, downregulation of other apico-basal polarity determinants, aPKC, Par6, and Par3, also leads to cilia loss (Fan *et al.* 2004; Sfakianos *et al.* 2007). To explain these observations in mechanistic terms, it has been postulated that Par proteins bridge transmembrane Crumbs 3 with a subunit of the main ciliary kinesin, Kif3a (Sfakianos *et al.* 2007).

As morpholino knockdown results are frequently difficult to interpret (Kok *et al.* 2015), we chose to analyze the role of *crumbs* in ciliogenesis using mutants of several zebrafish *crumbs* genes. We found that mutant alleles of *oko meduzy* (*crb2a*), *crb2b*, and *crb3a*, cause changes in cilia length. This is accompanied by a massive accumulation of other Crumbs proteins and intraflagellar transport (IFT) particle components in the ciliary compartment of *ome* and ome;*crb3a* mutants. Consistent with the above, *Crb3* knockdown in mammalian inner medullary collecting duct cells (IMCD3) cells affects the dynamics of IFT particle movement. These studies reveal *crumbs*-dependent mechanisms that affect the subcellular localization of Crumbs proteins and show that *crumbs* genes affect ciliary protein composition and modulate intraflagellar transport.

## Materials and Methods

### Zebrafish strains and maintenance

*crb3a* mutant alleles *crb3a^sh410^* and *crb3a^sh346^* were generated using transcription activator-like effector nucleases (TALENs) as described previously (Zu *et al.* 2013; Pooranachandran and Malicki 2016). The *crb2a^m98^* mutant allele was described previously in detail (Malicki *et al.* 1996; Malicki and Driever 1999; Omori and Malicki 2006) and the *crb2b^sa18042^* allele was obtained from the Sanger Institute TILLING project. Zebrafish were maintained in accordance with UK Home Office regulations and the UK Animals (Scientific Procedures) Act 1986. Fish genotypes were determined by fin-clipping adults at 3 months of age or later followed by DNA isolation, PCR amplification of mutant sites, and Sanger sequencing. The following primers were used: 5′-TTCTACACTTCTGGCTTCCG-3′ and 5′-ATTGTGGCCATCGTTGTA-3′ for *crb3a^sh410^* and *crb3a^sh346^*, and 5′-AAACTTCCGACTCCTCTCCG-3′ and 5′-AAAGATGTCCTACCCAGCTT-3′ for *crb2b^sa18042^*. During phenotypic analysis, mutants were compared to phenotypically wild-type siblings or to phenotypically wild-type animals derived from common ancesteral generation. Mutations that do not cause lethality (*crb3a^sh410^*; *crb3a^sh346^*; and *crb2b^sa18042^*) were maintained as homozygous strains. Consequently, analysis of cilia phenotype was performed on maternal/zygotic mutants.

### Photography of adult zebrafish

To record adult phenotypes, zebrafish 6 months old or older more were placed in weighing boats (7 ml, 611–9179; VWR) containing E3 medium with tricaine (E10521, 0.2 mg/ml; Sigma). Photographs were obtained using an iPAD Pro digital camera, 12MP, F/2.2, 29mm, phase detection autofocus.

### Immunostaining, mounting, and microscopy

Staining of whole zebrafish at 36 h postfertilization (hpf), 72 hpf, and 5 days postfertilization (dpf) was performed as previously described (Leventea *et al.* 2016). The following primary antibodies and dilutions were used: anti-acetylated tubulin, 1:500–1:1000 (T6793; Sigma [Sigma Chemical], St. Louis, MO); anti-CRB (Omori and Malicki 2006), 1:250; anti-Kif17(ab11261; Abcam), 1:500; anti-IFT88, 1:500; and anti-IFT52, 1:500. Anti-IFT antibodies were kindly provided by Brian Perkins. Embryos were then counterstained with DAPI to visualize nuclei. Stained embryos were placed in imprinted wells created by placing molds onto a liquid 1% agarose layer in 35-mm petri dishes (Leventea *et al.* 2016). To examine the cilia of the ear, the nasal pit, the pronephros, and the lateral line, embryos were positioned on their sides in the imprinted wells and immobilized by overlaying with 1.5% low-melting point agarose. Images of whole embryos were collected using an Olympus FV1000 confocal microscope with either a 40×/0.8 or 60×/0.9 water dipping lens.

### IMCD3 cell culture and siRNA experiments

Mouse inner medullary collecting duct cells stably expressing IFT88 (IMCD3-IFT88-GFP cells, a gift from Hiroaki Ishikawa) were grown in full medium containing Dulbecco’s modified Eagle’s medium (DMEM) and Ham’s F12 nutrient mixture (Invitrogen, Carlsbad, CA) supplemented with 10% fetal bovine serum (FBS) and 1% Pen/Strep Amphotericin B (100×) (Lonza) at 37° in a tissue culture incubator (Sanyo inCu Safe). Cells were seeded in cell culture flasks (cat no. 156472; Nunc). Medium was changed daily.

ON-TARGET plus SMART pool siRNAs (Dharmacon, GE Healthcare) against mouse *Crb3* were used to transfect IMCD3-IFT88 cells using Lipofectamine RNAiMAX transfection reagent (Life Technologies) following the manufacturer’s recommendations. The following siRNA target sequences were used: 5′-GCACCGGACCCUUUCCAA-3′, 5′-AGGCAAGCAGGAUGGGACU-3′, 5′-CAACACCCUCUUUGGGCAA-3′, and 5′-GAUAGGGACAAUAAAGGUU-3′. As a negative control, we used nontargeting pool directed to the following sequences: 5′-UGGUUUACAUGUCGACUAA-3′, 5′-UGGUUUACAUGUUGUGUGA-3′, and 5′-UGGUUUACAUGUUUUCUGA-3′, 5′-UGGUUUACAUGUUUUCCUA-3′.

### Immunostaining of IMCD3 cells

For staining with anti-acetylated tubulin and anti-Crumbs (CRB) antibodies, cells were rinsed with PBS, fixed with 4% PFA for 10 min at room temperature (RT), permeabilized with 0.1% Triton X-100 in PBS for 10 min, and blocked with 3% BSA in PBS (1×) for 30 min at RT. Cells were then incubated with appropriate primary and secondary antibodies using standard protocols. Cells were counterstained with DAPI and mounted on glass slides using ProLong Gold antifade reagent (Life Technologies). Imaging was performed on an Olympus FV1000 confocal microscope using a 60×/1.42 Plan Apo N oil lens.

### Total Internal Reflection Fluorescence (TIRF) imaging of IFT in IMCD3-IFT88 cells

IMCD3 cells were seeded on transwell cups (Costar, Cambridge, MA; 6.5 mm–0.5 μm pore size) at a density of 2 × 10^5^ cells/ml as previously described (Ott and Lippincott-Schwartz 2012; Ishikawa and Marshall 2015). Upon reaching 60–70% confluency, cells were transfected with siRNAs as above. 48 hr after transfection, cells were serum-starved for an additional 48 hr to induce ciliogenesis. The transwell cups were then placed in glass-bottom dishes (cat. no. 81153; ibidi) and imaged on an Eclipse Ti Microscope (Nikon, Shinagawa, Tokyo, Japan) supplied with a heating chamber (Oko Touch) using the Apo TIRF 100×, 1.49 NA oil lens (Nikon). Images were acquired at 100-msec intervals using the ixOn Ultra EMCCD camera (Andor Technology) and analyzed using the “KymoResliceWide” Fiji plug-in as previously described (Ishikawa and Marshall 2015). The lengths of tracks were measured using the “segmented line tool” in Fiji and expressed as the percentage of cilia length.

### Cilia length measurements and statistical analysis

Zebrafish cilia were measured on TIFF files of Z-stack projections of cristae and the nasal pit confocal images using the “segmented line tool” in ImageJ software. At least 10 animals (mutants and wild-types each) from two to three independent experiments were used. Measurements from each crista were averaged before performing comparisons. Cilia of IMCD3 cells were measured on TIFF images of cells stained with anti-acetylated tubulin antibody by tracing their length using ImageJ/FIJI software as above. Statistical analysis was carried out using the Student’s *t*-test, and the Mann–Whitney test included in GraphPad Prism 7.0 software (http://www.graphpad.com/). Data are presented as mean ± 95% C.I. Statistical significance is indicated as follows: * for *P* < 0.05, ** *P* < 0.01, *** *P* < 0.001, and **** *P* < 0.0001.

### Data availability

All animal strains and reagents will be distributed through international stock centers or directly by the Malicki laboratory.

## Results

### crb3a affects cilia length in vestibular system cristae

**Figure 1:**
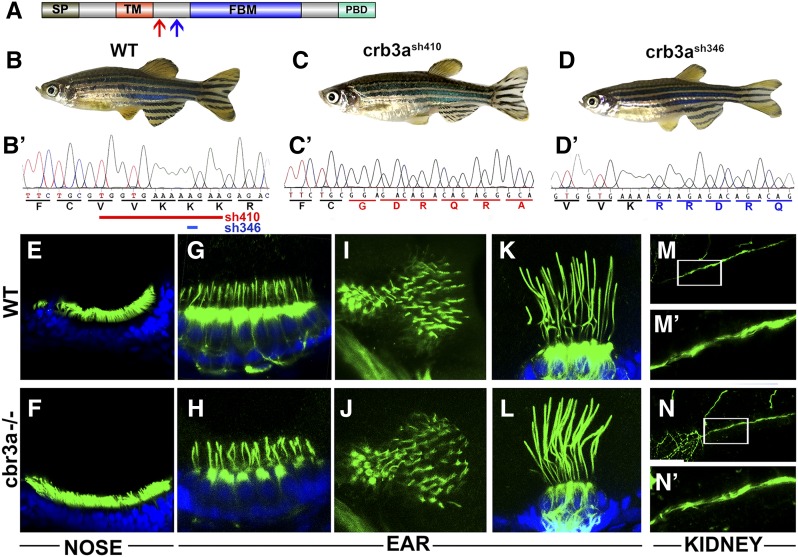
*crb3a* mutant phenotype. (A) Schematic of Crb3a protein domain structure. Signal peptide (SP); transmembrane domain (TM); FERM-binding motif (FBM); and PDZ-binding domain (PBD) are indicated. Red arrow indicates the start of the frameshift in *crb3a−/−^sh410^* mutant allele; blue arrow, the start of frameshift in *crb3a−/−^sh346^* allele. (B–D) External phenotypes of wild-type (WT) (B), *crb3a−/−^sh410^* homozygous mutant (C), and *crb3a−/−^sh346^* homozygous mutant (D) adult zebrafish. (B’–D’) Sequences of wild-type (B’), and two mutant alleles: *crb3a−/−^sh410^* (C’), and *crb3a−/−^sh346^* (D’). Deletions in *crb3a−/−^sh410^* (red line) and *crb3a−/−^sh346^* (blue line) mutants are indicated in (B’). (E–N’) Images of wild-type and *crb3a*−/− mutant embryos stained with anti-acetylated tubulin antibody (in green) and counterstained with DAPI (in blue) at 5 days postfertilization: olfactory placode (E and F); anterior macula (G and H); posterior macula (I and J); lateral crista (K and L); and pronephros (M and N). (M’ and N’) are enlarged images of pronephric cilia shown in (M and N).

Knockdown of the *CRB3* gene in MDCK cells was shown to block cilia formation (Fan *et al.* 2004). Similar phenotypes were seen following knockdowns of other apico-basal polarity determinants: aPKC, Par6, and Par3 (Fan *et al.* 2004; Sfakianos *et al.* 2007). Moreover, morpholino knockdown of *crb3a* in zebrafish was shown to reduce cilia length (Omori and Malicki 2006); However, these ciliary phenotypes have not been investigated in mutants. To address this deficiency, we generated several *crb3a* mutant alleles using TALEN nucleases as previously described (Zu *et al.* 2013). Two alleles were analyzed in this study, *crb3a^sh410^* and *crb3a^sh346^*. The former introduces a deletion of 13 bp, causing a frameshift between the TM and FERM domains (Figure 1A, red arrow and Figure 1C’). The latter contains a 1-bp deletion that also causes a frameshift at another site between the TM and FERM domains (Figure 1A, blue arrow and Figure 1D’). Homozygous carriers of either allele do not display any external abnormalities, survive to adulthood, and are fertile (Figure 1, B–D). This is also true for homozygous animals that originate from homozygous mothers and thus did not receive maternal contribution during embryogenesis.

**Figure 2:**
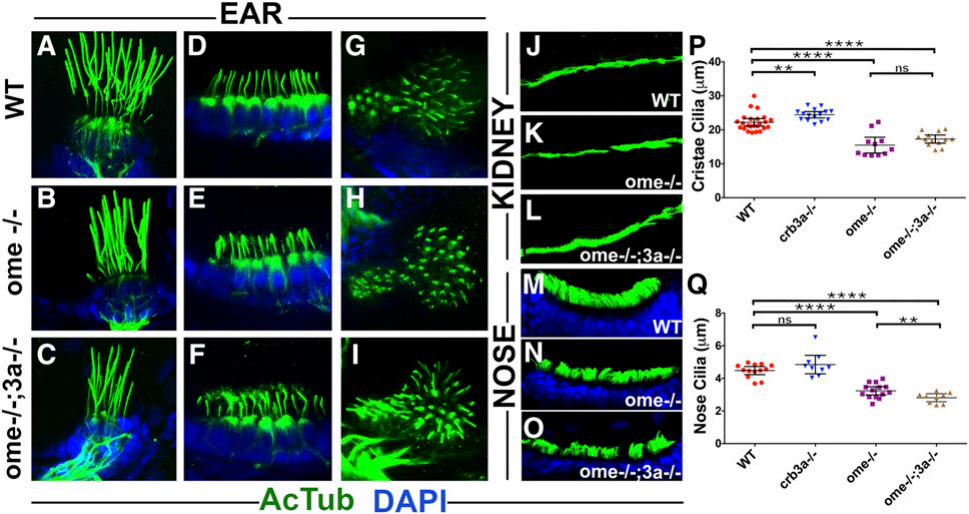
*oko meduzy* and *crb3a* genes modulate cilia length. (A–O) Whole-mount immunostaining of wild-type (WT), *ome−/−*, and *ome−/−*;*3a−/−* double mutant cilia in several tissues at 5 days postfertilization. (A–C) lateral crista, (D–F) anterior macula, (G–I) posterior macula, (J–L) pronephros, and (M–O) nasal pits. Zebrafish larvae were immunostained using anti-acetylated tubulin antibody (in green) and counterstained with DAPI (in blue) to visualize nuclei. (P) Graph of cilia length in the cristae of WT and *crumbs* mutants as indicated. Each dot represents the average length of all cilia in one crista. (Q) Graph of cilia length in the olfactory placodes of WT and *crumbs* mutants as indicated. In (P and Q), data were collected from at least two independent experiments using at least five animals per experiment. The mean and 95% C.I. are indicated. Based on Student’s *t*-tests; ** *P* < 0.01, *** *P* < 0.001, and **** *P* < 0.0001; not significant, ns. All differences were also significant based on Mann–Whitney test.

To assess cilia morphology in these mutants, we immunostained embryos at 5 dpf using anti-acetylated tubulin antibody. Mutant homozygotes for either allele do not manifest any gross ciliary phenotypes in nasal pits (Figure 1, E and F), anterior and posterior maculae (Figure 1, G–J), lateral cristae (Figure 1, K and L), or pronephroi (Figure 1, M–N’). However, thorough measurements of cilia length in vestibular system cristae revealed that *crb3a* mutant cilia are somewhat longer compared to those of the wild type (Figure 2P). These observations lead to the conclusion that *crb3a* function contributes to cilia length.

### oko meduzy (ome) mutations affect ciliogenesis

*ome* (*crb2a*) functions in the apico-basal polarity of the eye neuroepithelium and retinal neurogenesis. *ome* mutants are characterized by abnormal body axis curvature, edema, nonuniform eye pigmentation, grossly disorganized retinal neurons, and lethality by 7 dpf (Malicki *et al.* 1996; Malicki and Driever 1999; Omori and Malicki 2006). Previous studies did not evaluate *ome* function in cilia. To investigate the cilia phenotype, we analyzed vestibular cristae in *ome* mutants and found that cilia are shortened by ∼30% (Figure 2B, quantified in Figure 2P). To test functional relationship between *crb3a* and *ome*, we examined cilia of *ome*;*crb3a* double mutants. This analysis revealed that cilia of double mutants display similar length reduction to *ome* cristae, revealing that *ome* is epistatic to *crb3a*. Similarly, nose cilia are significantly shorter in both *ome* homozygotes and *crb3a*;*ome* double mutants when compared to those of the wild-type or *crb3a* homozygotes, (Figure 2, M–O, quantified in Figure 2Q). Moreover, olfactory placode cilia of *crb3a*;*ome* double mutants are significantly shorter than those of *ome* mutants (Figure 2Q). Cilia in kidney (Figure 2, K and L, compare to Figure 2J) and maculae (Figure 2, E, F, H, and I compare to Figure 2, D and G) are not obviously affected in *ome* or double mutants. These observations reveal that *ome/crb2a* and *crb3a* are necessary for proper cilia formation and control their lengths in a subset of tissues. It appears that *ome* functions downstream or in parallel to *crb3a* in cristae. These two genes also display some functional redundancy in nasal cilia.

### Crumbs proteins accumulate in cilia of oko meduzy mutants

Crumbs 3 localizes to cilia in mammalian cell culture and is enriched at the base of cilia in zebrafish (Fan *et al.* 2004; Omori and Malicki 2006). To investigate how *crumbs* mutations affect Crumbs protein localization, we immunostained embryos with anti-acetylated tubulin and anti-Crumbs antibodies. The anti-Crumbs antibody used in these experiments is directed to the cytoplasmic tail and recognizes all zebrafish Crumbs proteins on western blots of Crumbs-GST fusions (Hsu *et al.* 2006; Omori and Malicki 2006).

**Figure 3:**
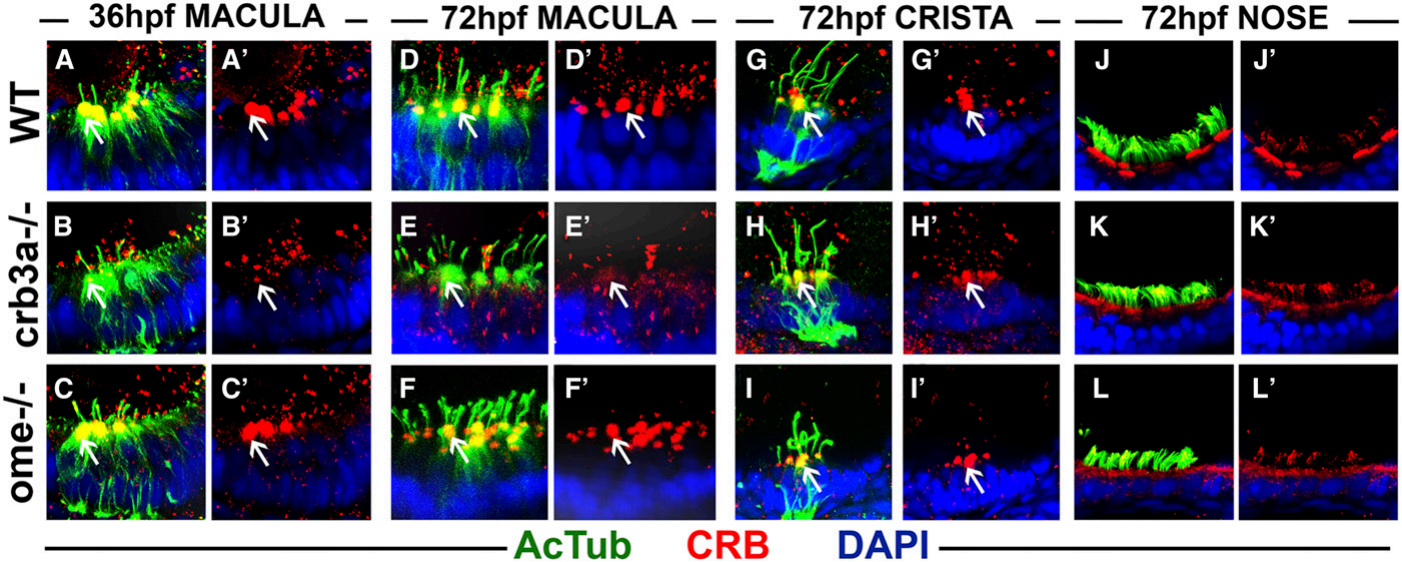
Crumbs expression in *crb3a* mutants at early stages of development. Confocal images of whole-mount cilia staining with anti-acetylated tubulin (AcTub) (green) and anti-CRB antibody (red). (A–C’) Staining of the otic vesicle at 36 h postfertilization (hpf). Crumbs proteins localize to the cilia base in the wild-type (WT) (A and A’) and *ome−/−* mutants (C and C’), but are absent in *crb3a−/−* mutant homozygotes (B and B’). At 72 hpf, Crumbs proteins still localize to the cilia base in maculae of WT animals (D and D’) and *ome−/−* mutants (F and F’), but very little signal is seen in *crb3a−/−* mutants (E and E’). No obvious differences are found in the localization of Crumbs proteins in the cristae of *ome−/−* (I and I’), *crb3a−/−* (H and H’), and WT individuals (G and G’). The localization patterns of Crumbs proteins in nasal pits of WT (J and J’), *crb3a−/−* mutant (K and K’), and *ome−/−* mutant (L and L’) animals do not show any obvious differences either. All samples were counterstained with DAPI to visualize nuclei. Arrows point to Crumbs signal at the apical surface of hair cells.

In the wild-type, Crumbs proteins are found at the base of hair cell kinocilia in ear maculae at 36 and 72 hpf (Figure 3, A, A’, D, and D’). This is no longer the case in *crb3a* mutant homozygotes (Figure 3, B, B’, E, and E’). However, in contrast to *crb3a* mutants, Crumbs localization in *ome* mutants is not affected (Figure 3, C, C’, F, and F’). These observations are in agreement with our previous report that *crb3a*, but not other *crumbs* genes, is strongly transcribed in the otic vesicle between 24 and 72 hpf (Omori and Malicki 2006). Crumbs proteins are also present at the apical surface of cells in olfactory placodes (Figure 3, J and J’). The apical localization is not obviously affected in either *crb3a* or *ome* mutants (Figure 3, K–L’).

**Figure 4:**
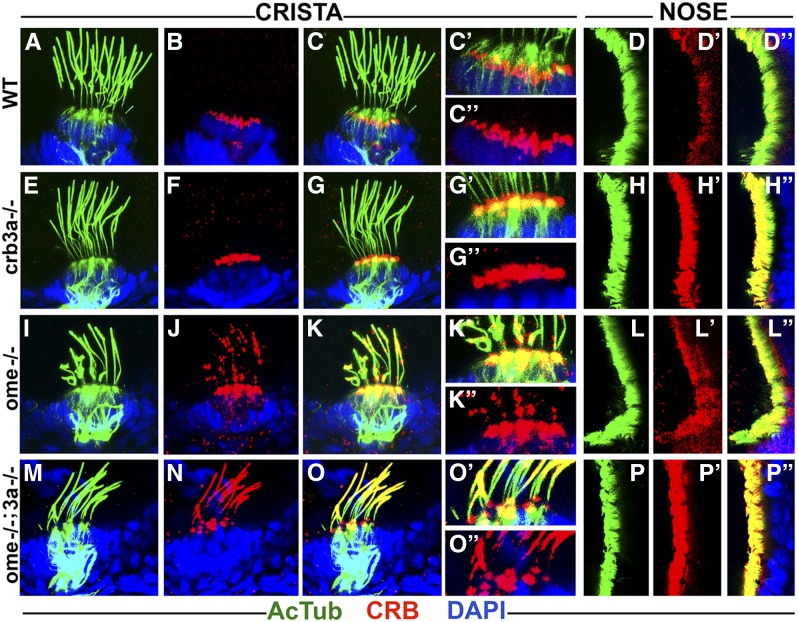
Crumbs proteins accumulate in cilia of *ome* mutants at 5 days postfertilization (dpf). Confocal images of whole-mount cilia staining with anti-acetylated tubulin antibody (AcTub) (green) and anti-CRB antibody (red) at 5 dpf. Shown are cristae and olfactory placodes of wild-types (WT), *crb3a−/−*, *ome−/−*, and *ome−/−*;crb*3a−/−* mutants as indicated. No obvious difference is seen in the localization of Crumbs proteins in cristae cilia of *crb3a−/−* mutants (E–G’’) when compared to WT (A–C’’). Crumbs proteins are strongly enriched in the cilia of nasal pits in *crb3a−/−* mutants (H–H’’) when compared to the WT (D–D’’). *ome−/−* and *ome−/−*;crb*3a−/−* double mutants show massive accumulation of Crumbs proteins inside cilia of cristae (I–K’’ and M–O’’) and nasal pits (L–L’’ and P–P’’). All samples were counterstained with DAPI to visualize nuclei (in blue).

In contrast to ear maculae, hair cells of semicircular canals contain normal levels of Crumbs in *crb3a* and *ome* mutant homozygotes at 3 dpf (Figure 3, H–I’ compare to Figure 3, G and G’). This is also the case for *crb3a* mutants at 5 dpf (Figure 4, E–G’’, compare to Figure 4, A–C’’). However, we did observe strong enrichment of Crumbs proteins in olfactory placode cilia of these mutants (Figure 4, H–H’’, compare to Figure 4, D–D’’, 10/10 olfactory placodes). Interestingly, in *ome* mutant homozygotes, Crumbs proteins are mislocalized into cilia of both ear cristae and olfactory placodes (Figure 4, I–K’’, 23/23 cristae and Figure 4, L–L’’, 11/14 olfactory placodes). These findings reveal that *ome*, and to a lesser extent *crb3a*, strongly affect the subcellular localization of other Crumbs proteins. An enrichment of Crumbs proteins is also found in *ome*;*crb3a* double mutants both in cristae and olfactory placodes (Figure 4, M–O’’ 33/33 cristae and Figure 4, P–P’’ 6/6 olfactory placodes). While Crumbs staining forms puncta in *ome* mutant cilia, *ome*;*crb3a* double mutants display a uniform Crumbs signal along most of the ciliary axoneme, with the exception of the proximal region (Figure 4, I–K’’, compare to Figure 4, M–O’’). These findings reveal regulatory relationships between *crumbs* genes.

### IFT proteins are highly enriched in cilia of ome;crb3a double mutants

**Figure 5:**
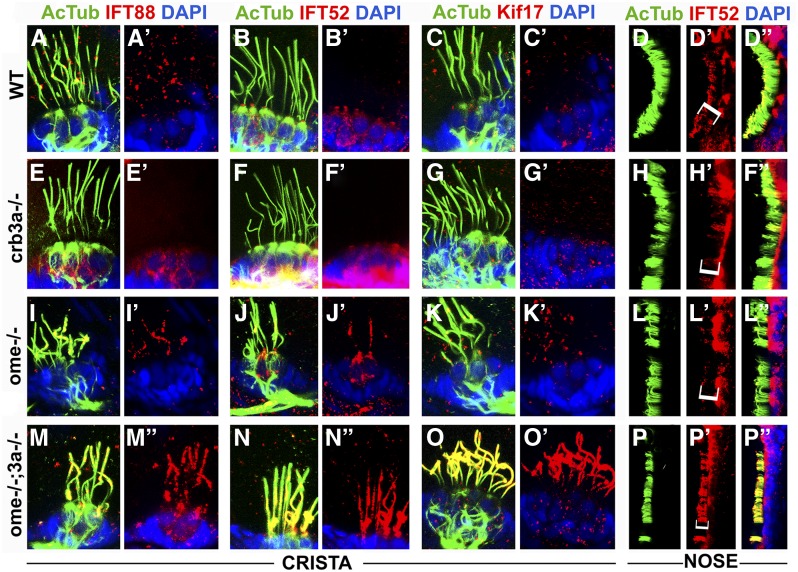
Intraflagellar transport (IFT) particle components accumulate in the ciliary compartment of *crumbs* mutants. Confocal images of whole-mount cilia staining with antibodies to acetylated tubulin (AcTub) (green), and to IFT proteins (in red): IFT88, IFT52, and Kif17 at 5 days postfertilization. Shown are cristae and olfactory placodes of wild-type (WT), *ome−/−*, *crb3a−/−*, and *ome−/−*;*crb3a−/−* double mutants as indicated. IFT proteins are not detected in the ciliary shaft of WT cristae using this staining method (A–C’) and a low amount of IFT52 is found in WT olfactory cilia (D–D’’). Similarly, in *crb3a−/−* mutants, IFT proteins are not detected in cilia (E–H’’). Low levels of some IFT proteins are found in cristae cilia of *ome−/−* mutants (I–K’). Compared to WT, IFT52 localization is not affected in olfactory placode cilia of ome−/− mutants (L–L’’). In contrast to that, IFT proteins, including Kif17, strongly accumulate in cilia of *ome−/−*;*crb3a−/−* double mutants (M–P’’). All samples were counterstained with DAPI to visualize nuclei (in blue). Brackets in (D’, H’, L’, and P’) indicate nasal cilia.

Par3, a key regulator of apico-basal cell polarity, is required for ciliogenesis in cell culture conditions (Sfakianos *et al.* 2007). Furthermore, the Par3 C-terminal region binds directly to Kif3a, the main anterograde motor of IFT particles (Nishimura *et al.* 2004). This led to the hypothesis that the Par3/Par6/aPKC complex bridges Crumbs proteins and Kif3a (Sfakianos *et al.* 2007). If true, Crumbs proteins could affect IFT by competing for the Kif3A motor. To test whether the loss of *ome* and/or *crb3a* function and the accompanying accumulation of Crumbs proteins in cilia affects IFT, we stained *ome* mutants and *ome*;*crb3a* double mutants with antibodies to IFT particle components: IFT88, IFT52, and Kif17. In *crb3a* mutants, IFT protein localization in cilia is largely unchanged (Figure 5, E–H’’, compare to Figure 5, A–D’’). However, weak accumulation of IFT proteins is observed in cristae cilia of *ome* mutants (Figure 5, I–K’). The olfactory cilia in both *crb3a* and *ome* single mutants do not show obvious differences in IFT distribution when compared to the wild type (Figure 5, H–H’’ and L–L’’). Strikingly, in *ome*;*crb3a* double mutants, IFT proteins massively accumulate inside cilia of ear cristae (Figure 5, M–O’) and the olfactory placode (Figure 5, P–P’’). In a control experiment, we have not observed any enrichment of γ-tubulin in *ome*;*crb3a* double mutants (data not shown). These observations indicate that *ome* and *crb3a* genes function redundantly in the ciliary localization of IFT proteins.

### CRB3 affects IFT train dynamics in IMCD3 cells

**Figure 6:**
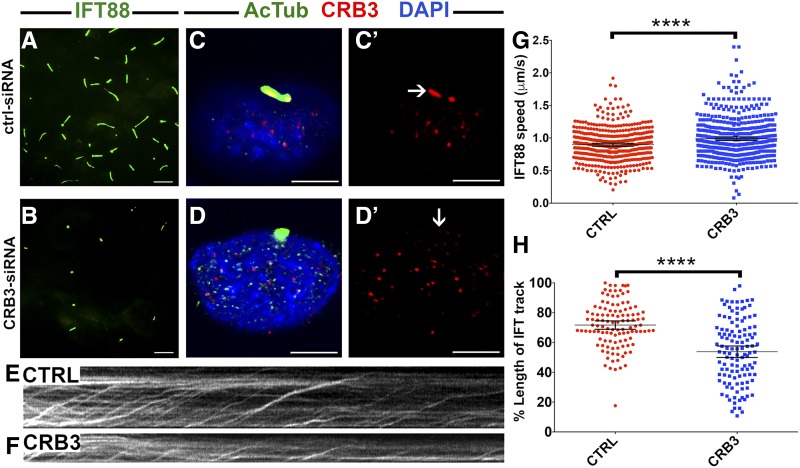
CRB3 knockdown in mammalian cells affects intraflagellar transport (IFT) dynamics. (A and B) Maximum projections of total internal reflection fluorescence (TIRF) time-lapse recordings of IMCD3 cells grown on transwells and transfected with scrambled Ctrl-small interfering RNA (siRNA) (A) or CRB3-siRNA (B). These cells are stably transfected with an IFT88-GFP construct to visualize intraflagellar transport (green signal). (C–D’) Confocal images of control (CTRL) siRNA- (C and C’) and CRB3 siRNA-treated (D and D’) IMCD3 cells. Cilia are stained with antibodies to acetylated tubulin (AcTub) (green) and Crumbs (red). Samples are counterstained with DAPI to mark nuclei (in blue). (E and F) Kymographs of IFT movement in an IMCD3-IFT88 cell line transfected with CTRL or CRB3 siRNA as indicated. (G) Graph showing the speed of IFT particle movement in cilia of IFT88-GFP IMCD3 cells. Date collected from three independent experiments. (H) Lengths of IFT tracks in CTRL siRNA- and CRB3 siRNA-transfected cells expressed as percentages of total cilia length. Data are collected from two independent experiments. In (G and H), the mean and 95% C.I. are indicated. *P* < 10^−4^ based on Student’s *t*-tests. Bar, 10 μm (A and B) and 5 μm (C–D’).

To further the understanding of the relationship between Crumbs proteins and intraflagellar transport, we decided to test whether IFT train movement is affected by *crumbs* genes. Imaging of IFT movement is difficult in zebrafish but can be efficiently performed in mammalian cells (Jin *et al.* 2014; Ishikawa and Marshall 2015). To this end, we knocked down CRB3 in an IMCD3 cell line stably expressing an IFT88-GFP fusion (Ishikawa and Marshall 2015). As reported previously, CRB3-knockdown cells display fewer and shorter cilia compared to controls (Figure 6, A–D) and the level of CRB3 proteins is reduced (Figure 6, C’ and D’). Imaging of IFT particle movement using IFT88 fluorescence (Figure 6, E and F) revealed that IFT particle speed is somewhat faster in knockdown cells, compared to controls (Figure 6G). Moreover, when adjusted for cilia length, IFT tracks are 25% shorter in knockdown cells when compared to control cells (Figure 6H). These observations are consistent with the idea that Crumbs affects IFT processivity and speed.

### crb2b mutation increases cilia length in a subset of tissues

**Figure 7:**
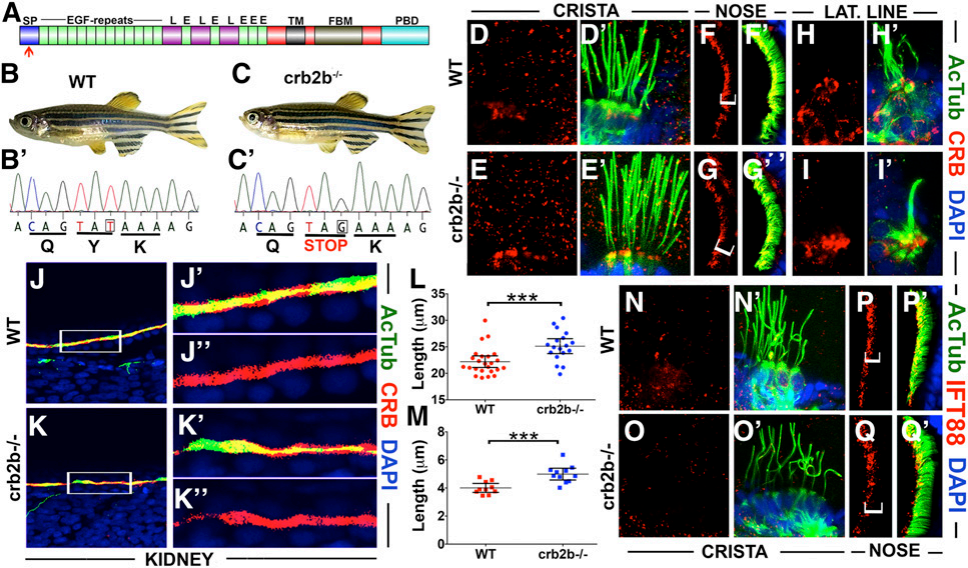
*crb2b* affects cilia length. (A) Schematic of Crb2b protein domain structure (not to scale). Indicated are the signal peptide (SP), EGF-like repeats (E), Laminin G domains (L), transmembrane domain (TM), FERM-binding motif (FBM), and PDZ-binding domain (PBD). Red arrow shows the position of mutation in the *crb2b−/−^sa18042^* mutant allele. (B and C) Phenotypes of wild-type (WT) (B) and *crb2b−/−^sa18042^* (C) homozygous mutant adult zebrafish. (B’ and C’) Sequences of WT and *crb2b−/−^sa18042^* mutant alleles. (D–I’) Whole-mount staining of WT and *crb2b−/−* mutants using anti-acetylated tubulin (AcTub) (green), and anti-CRB (red) antibodies at 5 days postfertilization. Samples were counterstained with DAPI to visualize nuclei (in blue). Crumbs proteins are not detected in the cilia of cristae (D–E’) and the lateral (LAT.) line (H–I’) of *crb2b−/−* mutants or their WT siblings. No differences in Crumbs signal are found between WT and mutants in the cilia of olfactory placodes (F–G’) and the pronephric duct (J–K’’). (L) Graph of cilia length in WT and *crb2b−/−* mutants. Each dot represents the average length of all cilia in one crista. Data were collected from three independent experiments using at least five animals per experiment. (M) Graph of cilia length in olfactory placodes of WT and *crb2b−/−* mutants. (N–O’) IFT proteins are not detected in the cristae cilia of WT (N and N’) and *crb2b−/−* mutants (O and O’). (P–Q’) IFT88 localization is not obviously different in nasal pit cilia of WT (P and P’) and *crb2b−/−* mutant (Q and Q’) animals. Brackets in (F, G, P, and Q) indicate nasal cilia. In (L and M), the mean and 95% C.I. are indicated. *P* < 0.001 based on Student’s *t*-test and Mann–Whitney test.

Morpholino knockdown studies of *crb2b* revealed that this gene is necessary for the elongation and motility of pronephric cilia (Omori and Malicki 2006). To gain further insight into *crb2b* function in cilia formation, we analyzed homozygous carriers of the *crb2b^sa18042^* allele. The zebrafish *crumbs 2b* gene encodes two polypeptides that share most of the amino acid sequence. The shorter polypeptide does not include 11 N-terminal fibroblast growth factor-like repeats present in the long form and features a separate signal sequence (Zou *et al.* 2012). The *crb2b^sa18042^* allele that we chose to use contains a stop codon at amino acid 10 of the long form, and thus is likely to eliminate the function of the long form (red arrow in Figure 7, A and B’–C’). A possible use of an alternative initiation codon at position 19 of the open reading frame could lead to protein expression, but it would eliminate most of the signal sequence rendering the long form of Crb2b dysfunctional. *crb2b^sa18042^* homozygotes have normal external appearance and are fertile (Figure 7, B and C). This is also true for the offspring of homozygous mothers. To analyze cilia morphology in these mutants, we stained them using anti-acetylated tubulin antibody as above. No gross abnormalities were seen in the cilia of most tissues, including the inner ear (Figure 7E’, compare to Figure 7D’), the olfactory placode (Figure 7G’, compare to Figure 7F’), and the lateral line (Figure 7I’, compare to Figure 7H’). Similarly, contrary to the phenotype observed following morpholino knockdown (Omori and Malicki 2006) (Table 1), pronephric cilia were not obviously affected in *crb2b* mutants when compared to the wild type (Figure 7, K and K’, compare to Figure 7, J and J’). Staining of mutant homozygotes with anti-CRB antibody did not reveal differences in Crumbs protein localization in cristae (Figure 7E, compare to Figure 7D), olfactory placodes (Figure 7G, compare to Figure 7F), the lateral line (Figure 7I, compare to Figure 7H), and the pronephros (Figure 7K’’, compare to Figure 7J’’). However, a statistically significant difference of cilia length was observed between *crb2b* mutants and the wild-type in cristae and the olfactory placode (Figure 7, L and M). Contrary to cilia shortening seen in *ome* and *ome*;*crb3a* double mutants, and similar to the *crb3a* cilia phenotype (Figure 2P), cilia of *crb2b* mutant homozygotes are longer than those of their wild-type siblings. To test whether IFT protein localization is affected in these mutants, we immunostained homozygous *crb2b* mutant embryos for IFT88 at 5 dpf. No detectable difference was seen in the localization of IFT88 between *crb2b* mutants and the wild-type in inner ear cilia (Figure 7O, compare to Figure 7N) and the nose (Figure 7Q, compare to Figure 7P). These results further confirm that *crumbs* genes modulate cilia lengths in several tissues. In contrast to *ome* mutants, *crb2b^sa18042^* mutant homozygotes do not display crumbs upregulation in cristae cilia.

**Table 1:** Summary of mutant and morphant *crumbs* phenotypes in cilia

Genotype	Tissue Examined	Cilia Length Phenotype	Crumbs Localization (3 dpf)	Crumbs Localization (5 dpf)	IFT in Cilia (5 dpf)
Wild-type	Ear macula	—	Cilia base	Cilia base	n.d
	Ear cristae	—	Cilia base	Cilia base	None
	Nasal placode	—	Cilia base	Weak in ciliary shaft	?
	Kidney	—	Cilia base/apical surface (36 hpf)	n.d.	n.d.
*crb3a−/−*	Ear macula	n.d.	Absent	Cilia base	n.d.
	Ear cristae	Longer	Cilia base	Cilia base	None
	Nasal placode	No change	Cilia base	Strong ciliary shaft	?
*crb3a* MO	Ear macula	Shorter*^a^*	Reduced (2 dpf)*^a^*	n.d.	n.d.
*ome−/−*	Ear macula	n.d.	Cilia base	Cilia base	n.d.
	Ear cristae	Shorter	Cilia base	Cilia base, puncta in ciliary shaft	Weak
	Nasal placode	Shorter	Cilia base	Ciliary shaft	Cilia base
*crb3a−/−*; *ome−/−*	Ear cristae	Shorter	n.d.	Cilia base, ciliary shaft, weaker proximally	Strong
	Nasal placode	Shorter	n.d.	Strong ciliary shaft	Ciliary shaft
*crb2b−/−*	Ear cristae	Longer	n.d.	Cilia base	None
	Nasal placode	Longer	n.d.	Weak in ciliary shaft	?
	Kidney	No obvious change	cilia base/apical surface	n.d.	n.d.
*crb2b* MO	Kidney	Shorter and disorganized*^a^*	reduced (1 dpf)*^a^*	n.d.	n.d.

Cilia length relative to wild-type cilia. dpf, days postfertilization; IFT, intraflagellar transport; n.d., not determined; ?, weak signal comparable to background; hpf, hours postfertilization. MO, morpholino.

a Omori and Malicki (2006)

## Discussion

Our studies reveal that *crumbs* genes function in three interconnected aspects of ciliogenesis: the regulation of protein composition in the ciliary shaft, IFT movement dynamics, and cilia length determination (summarized in Table 1). The absence of some *crumbs* genes, either singly and/or in double mutants, results in a massive accumulation of other Crumbs proteins and IFT particle components inside the ciliary shaft in some tissues. In a subset of cilia, the increase in ciliary Crumbs localization correlates with a decrease of cilia length. Interestingly, IFT dynamics is affected following *Crb3* knockdown in mammalian cells; IFT trains are somewhat faster and IFT tracks are markedly shorter. As discussed below, this may be related to a global role of Crumbs proteins in the morphogenesis of the apical surface of the cell.

An increase in ciliary Crumbs content in *ome* mutants is counterintuitive and reveals that *crumbs* genes or their protein products may negatively regulate each other. Such regulation could occur at the level of transcript or protein expression. It could also be mediated by protein degradation pathways. Previous studies suggested that *crumbs* expression may be regulated post-transcriptionally. The zebrafish Crb3a protein is enriched in mechanosensory hair cells while its transcript is uniformly expressed throughout the otic vesicle, suggesting a regulatory mechanism that affects translation or protein stability (Omori and Malicki 2006). Since mouse studies of the retina did not detect changes in the transcriptome of the *Crb2* mutant during development, cross talk between *crumbs* genes on the level of transcriptional regulation appears less likely (Alves *et al.* 2013). Consistent with the above, zebrafish studies did not reveal compensatory Crumbs protein upregulation in *ome* mutants and similarly did not detect transcriptional upregulation of *Crb2b* in the same mutants (Hsu *et al.* 2006). Alternatively, as discussed below, changes in Crumbs protein level in cilia may reflect the function of this group of genes in gating mechanisms at the cilia base.

Equally unexpected is the enrichment of IFT proteins in the cilia of *crumbs* mutants. It could be partially explained by the trapping of the heterotrimeric IFT kinesin by the mislocalized Crumbs in the ciliary shaft (see below). In addition, the regulation of IFT protein content by Crumbs could occur at the level of gating mechanisms that regulate trafficking into the ciliary compartment. This is suggested by observations that a Crumbs 3 isoform interacts with Importin β-1 in a RAN-regulated manner (Fan *et al.* 2007). A related importin, importin β-2 localizes to the proximal region of the ciliary axoneme and was proposed to mediate the ciliary entry of kif17, one of the two IFT kinesins, also in a RAN-regulated fashion (Dishinger *et al.* 2010). It is thus possible that Crumbs mutations affect RAN–Importin-mediated gating mechanisms at the cilia base that regulate IFT entry into the ciliary compartment. This possibility is also supported by observations that Crumbs is enriched at the base hair cell kinocilia, where it could function in regulating cilia-directed traffic (Omori and Malicki 2006).

*crumbs* mutants display cilia abnormalities only in some organs. One possible reason is that *crumbs* genes, *crb2b* and *crb3a* in particular, are expressed in a subset of tissues. Another and perhaps more intriguing possibility is that the *crumbs* cilia phenotype varies across tissues due to intrinsic differences in cilia assembly mechanisms. Cristae cilia in particular are genetically different from most other cilia. The most striking indication of their unique genetic characteristics is that they are unaffected in mutants of *kif3b*, a subunit of the major ciliary kinesin, while most other cilia, including kinocilia of ear maculae, are absent in *kif3b* mutants (Zhao *et al.* 2012). Similarly, the *kif3a* mutant phenotype of cristae cilia differs from that of other cilia. Short-cristae cilia form in the absence of *kif3a* function and, in contrast to maculae for example, IFT88 protein persists at the base of these cilia in *kif3a* mutants (Pooranachandran and Malicki 2016). Although morphological abnormalities of cilia in *crumbs* mutants are fairly subtle, the accumulation of Crumbs and IFT proteins in the ciliary shaft may have profound functional consequences, such as malfunction of cilia-mediated signal transduction cascades. This may account for the severity of the *oko meduzy* phenotype in many organs including the central nervous system, the cardiovascular system, and the pronephros (Malicki and Driever 1999; Omori and Malicki 2006).

What mechanism could account for the role of Crumbs in cilia elongation? It was previously reported that the C-terminus of Par3, a key regulator of apico-basal polarity, binds directly to the C-terminal coiled coil region of Kif3a, the main anterograde motor of IFT particles (Nishimura *et al.* 2004). This, combined with observations that Crb3 and Par3 function in ciliogenesis, led to the idea that the Par3/Par6/aPKC complex bridges Crumbs proteins to Kif3a (Fan *et al.* 2004; Sfakianos *et al.* 2007). A genetic interaction between *crumbs* and *kinesin-1* was also reported in the fly eye (League and Nam 2011). It is then tempting to hypothesize that Crumbs proteins compete for the Kif3a motor and, as a consequence, slow down IFT. In this model, cilia shortening in *ome* mutants is explained by the accumulation of other Crumbs proteins in cilia.

The results of *crumbs* function analysis in tissue culture are difficult to reconcile with cilia elongation in the zebrafish model. In contrast to fish phenotypes, RNAi knockdown in tissue culture has the opposite effect and causes cilia loss. This could be due to a global role of *crumbs* in cell polarity. Although cell junctions are largely intact in *Crb3* siRNA knockdown cells and in *Crb3* mutant mice, analysis of epithelia in *Crb3* mouse mutants reveals substantial abnormalities, such as the appearance of prominent blebs on the apical surface of lung cells and a shortening and fusion of apical villi in the intestine (Fan *et al.* 2004; Whiteman *et al.* 2014). These defects reveal a role of *Crb3* in apical surface morphogenesis, which could account for cilia loss in tissue culture studies. Nonetheless, the *Crb3* mouse mutant phenotype is inconsistent with tissue culture studies as it does not affect cilia morphology (Whiteman *et al.* 2014).

*crb3* mutant phenotypes also differ between fish and mice; the mouse knockout phenotype is lethal whereas the zebrafish *crb3a* phenotype is not (Whiteman *et al.* 2014). This is most likely due to the duplication of the *crb3* gene in the zebrafish genome. It has been argued for quite a while now that gene duplication frequently leads to a subfunctionalization of duplicates relative to the ancestral gene (Force *et al.* 1999; Braasch *et al.* 2016). This is likely to have happened in the case of *crb3* genes: zebrafish *crb3a* is mainly expressed in the otic vesicle and only weakly in the digestive system, while the *crb3b* transcript is found strongly expressed in the digestive system and not at all in the ear (Omori and Malicki 2006). It is thus likely that zebrafish *crb3a* mutants are viable because, in contrast to mouse *Crb3* mutants, they do not affect essential digestive organ functions.

Differences between the outcome of tissue culture studies and genetic analysis in animal models are not uncommon and are frequently difficult to explain. Although HDAC6 and Rab8 appear to function as potent regulators of ciliogenesis in tissue culture studies (Nachury *et al.* 2007; Pugacheva *et al.* 2007), mice mutant for these genes do not display cilia defects (Zhang *et al.* 2008; Sato *et al.* 2014; Ying *et al.* 2016). Similarly, substantial differences are frequently seen between morphant and mutant phenotypes in zebrafish (Kok *et al.* 2015). In our study of *crumbs* mutants, we also found phenotypic differences in comparison to morpholino knockdowns performed previously (Omori and Malicki 2006) (summarized in Table 1). Such differences could be explained by compensatory mechanisms that become active in mutants, such as the upregulation of paralogous genes. Increased presence of Crumbs proteins in the cilia of *crumbs* mutants may represent such a compensatory mechanism. Such mechanisms may account for some of the differences seen between tissue culture, morphant, and mutant analyses. Taken together, our data show that some *crumbs* genes affect the subcellular localization of protein products expressed by other *crumbs* genes, either through direct regulatory relationships or indirectly by affecting the function of gating mechanisms at the cilia base. *crumbs* genes function in multiple interrelated aspects of ciliogenesis, including intraflagellar transport, the determination of cilia length, and the protein composition of the ciliary shaft.
